# Federated CT foundation models for multi-center detection of lymph node metastasis in pancreatic cancer

**DOI:** 10.1038/s41598-026-47631-2

**Published:** 2026-04-09

**Authors:** Parinishtha Bhalla, David Dueñas Gaviria, Patrick Kupczyk, Ali Seif Amir Hosseini, Lena Conradi, Uli Fehrenbach, Matthaeus Felsenstein, Dou Ma, Alexander Semaan, Shadi Albarqouni

**Affiliations:** 1 Clinic for Diagnostic and Interventional Radiology, University Hospital Bonn, University of Bonn, Bonn, Germany; 2 Clinic and Polyclinic for General, Visceral, Thoracic, and Vascular Surgery, University of Bonn, Bonn, Germany; 3 Department of Clinical and Intervention Radiology, University Medical Center Göttingen, Göttingen, Germany; 4 Department of General, Visceral and Pediatric Surgery, University Medical Center Göttingen, Göttingen, Germany; 5 Department of Radiology, Charité-Universitätsmedizin Berlin, Berlin, Germany; 6 Department of Surgery, CCM | CVK, Charité-Universitätsmedizin Berlin, Berlin, Germany; 7 Department of General and Visceral Surgery, CBF, Charité-Universitätsmedizin Berlin, Berlin, Germany; 8 TUM School of Computation, Information and Technology, Technical University of Munich, Munich, Germany

**Keywords:** Foundation Models, Pancreatic ductal adenocarcinoma, Federated Learning, Cancer, Computational biology and bioinformatics, Mathematics and computing, Oncology

## Abstract

Pancreatic ductal adenocarcinoma (PDAC) remains one of the most lethal malignancies, with prognosis strongly influenced by the presence of lymph node metastasis (LNM). However, preoperative LNM assessment from computed tomography (CT) is limited by low sensitivity, high inter-observer variability, and substantial heterogeneity across imaging protocols. This retrospective multi-center study (546 patients from three institutions) introduces a privacy-preserving deep learning framework that integrates large-scale CT foundation model pre-training with heterogeneity-aware federated optimization to improve LNM detection in PDAC. A CT Vision Foundation Model, pre-trained on 148,000 volumetric CT scans using contrastive self-supervised learning, is fine-tuned to generate transferable 3D representations for patient-level LNM classification. To enable decentralized model training while mitigating inter-institutional variability, we extend federated aggregation to jointly account for label-distribution discrepancies and representation-level divergence across clients. The centralized model achieved a balanced accuracy of 0.601 and a diagnostic odds ratio (DOR) of 3.45, outperforming classical machine learning baselines and prior PDAC LNM approaches. Under federated settings, the proposed heterogeneity-aware strategy consistently outperformed standard FedAvg, recovering a substantial proportion of the centralized model’s performance while preserving strict data privacy. In particular, it improved balanced accuracy by 12.6% over FedAvg and demonstrated superior discriminative ability across all participating cohorts. These findings indicate that combining foundation model pre-training with discrepancy-aware federated learning enhances generalization, robustness, and clinical relevance for multi-center PDAC LNM detection. The proposed framework offers a scalable and privacy-preserving pathway for deploying deep learning models across distributed healthcare systems.

## Introduction

Pancreatic ductal adenocarcinoma (PDAC), which accounts for more than 90% of pancreatic cancer cases, remains one of the most lethal malignancies worldwide. Despite advances in surgical management, systemic therapy, and perioperative care, improvements in overall survival have been minimal^1^. Owing to its aggressive biology and the absence of early symptoms, PDAC is frequently diagnosed at an advanced or unresectable stage, making it the sixth leading cause of cancer-related mortality globally^2^. Improving early risk stratification and accurate staging therefore remains a critical clinical priority.

Lymph node metastasis (LNM) is among the strongest prognostic indicators in localized PDAC, with direct implications for survival, surgical decision-making, and the selection of neoadjuvant therapy^3^. However, reliable preoperative assessment of LNM remains highly challenging. Computed tomography (CT), the standard imaging modality for PDAC staging, relies primarily on nodal size and morphological criteria, which are known to exhibit poor sensitivity (14–44%), limited specificity, and substantial inter-observer variability^4,5^. These limitations frequently result in understaging and may lead to suboptimal therapeutic strategies^6^. Methods capable of more reliably detecting LNM from routine CT imaging could therefore offer substantial clinical value.

Deep learning (DL) methods have shown considerable promise in improving PDAC-related image analysis, including tumor detection, segmentation, and outcome prediction^7–9^. Recent DL-based approaches for LNM classification using contrast-enhanced CT have demonstrated improved diagnostic performance relative to size-based thresholds and radiomic models^10,11^. In a related domain, multi-center approaches to cancer detection have highlighted the importance of addressing inter-institutional heterogeneity^12^. Recent work by Zheng *et al.*^13^ proposed a deep attention learning framework that integrates lymph node segmentation, identification, and holistic metastasis status prediction, and demonstrated improved performance over radiomics and standard deep learning baselines in multi-center PDAC cohorts, highlighting the importance of anatomically informed modeling for this task. Our previous work^14^ also demonstrated that CNNs trained on multi-center CT data can identify LNM in PDAC with reasonable accuracy. However, as with most existing studies, model generalizability was constrained by limited cohort size and a lack of external validation. These constraints underscore the need for collaborative model development across institutions.

Federated learning (FL)^15^ offers a privacy-preserving paradigm for multi-center collaboration by enabling decentralized model training without sharing raw patient data ^16–18^. FL has been successfully applied in several medical imaging domains, including neuroimaging^19^ and chest CT analysis^20^. However, standard FL algorithms such as FedAvg implicitly assume similar data distributions across participating sites, an assumption rarely satisfied in clinical practice. Variations in scanner hardware, reconstruction protocols, contrast timing, and patient demographics introduce substantial heterogeneity, which can lead to biased local updates and unstable global convergence^21,22^. Effectively addressing non-IID heterogeneity remains a central challenge for real-world FL deployment in healthcare. While methods such as FedBN^23^ attempt to mitigate this domain shift by retaining site-specific normalization parameters locally, and FedALA^24^ employs adaptive local aggregation for client-side personalization, both approaches can remain vulnerable to catastrophic model collapse under severe feature and label discrepancies. Other lines of work have explored alternative strategies to address non-IID heterogeneity, including parameter-efficient fine-tuning^25^, prompt-based cooperation^26^, and genetic algorithm-enhanced convergence^27^, each offering complementary but partial solutions to the underlying distribution mismatch.

Recent advances in large-scale medical foundation models provide a promising avenue to mitigate these limitations. Foundation models pre-trained using contrastive or self-supervised learning on large, diverse CT repositories have demonstrated strong transferability and robustness across downstream tasks^28,29^. Their ability to capture domain-invariant representations makes them particularly attractive for federated settings, where heterogeneous data sources must be harmonized without direct data sharing.

In this work, we propose a federated deep learning framework for preoperative LNM prediction in PDAC that integrates foundation model transfer learning with heterogeneity-aware federated optimization. We employ the CT Vision Foundation Model^28^, pre-trained on more than 148,000 volumetric CT scans from the Imaging Data Commons (IDC), as a robust encoder for representation learning. To address cross-site variability, we adopt an adaptive aggregation strategy inspired by FedDisco^30^ and extend it by incorporating a representation-level discrepancy term that captures divergence in learned decision boundaries across clients. This design enables principled mitigation of both label-distribution imbalance and feature-level domain shift.

Compared with our earlier centralized approach^14^, the present study introduces a multi-institutional, privacy-preserving framework explicitly designed to address real-world clinical heterogeneity.

**Our main contributions are as follows:**

- We demonstrate that fine-tuning a large-scale CT foundation model improves LNM prediction performance in PDAC across heterogeneous clinical centers.
- We introduce a heterogeneity-aware FL framework that jointly accounts for label-distribution imbalance and representation-level divergence, leading to improved robustness and generalization under non-IID conditions (1).

## Methods

**Fig. 1:**
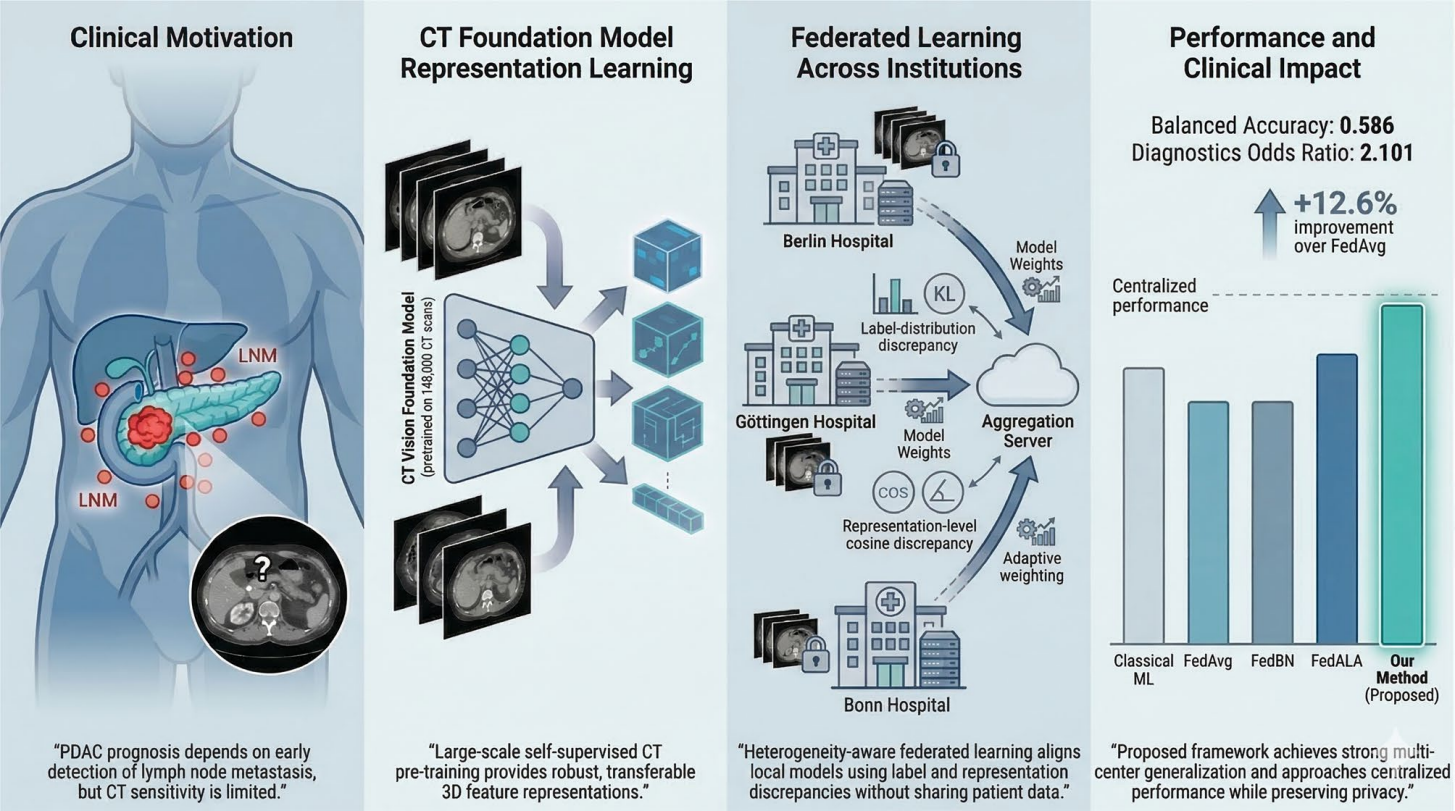
Overview of the proposed framework. A CT foundation model provides robust 3D representations for (LNM) detection in PDAC. Heterogeneity-aware FL enables collaborative training across institutions while preserving patient privacy and mitigating cross-site domain shift.

### Study cohort and data acquisition

This retrospective multi-center study included 546 patients diagnosed with PDAC from three German institutions: (1) University Hospital Bonn (UKB, $n=180$), (2) Charité – University Medicine Berlin ($n=163$), and (3) University Medicine Göttingen (UMG, $n=203$). All patients underwent contrast-enhanced abdominal CT imaging acquired in the portal-venous phase as part of routine clinical care. LNM was annotated at the patient level based on postoperative histopathology and/or multidisciplinary tumor board consensus.

Each institution independently partitioned its dataset into 70% training, 10% validation, and 20% testing subsets using stratified sampling. The prevalence of LNM-positive cases was 71% (UKB), 69% (Berlin), and 64% (Göttingen). All data were anonymized prior to analysis, and the study was conducted under institution-specific ethical approvals with informed consent waived due to its retrospective design.

### Image preprocessing

To standardize imaging inputs across institutions, all CT volumes were processed using a consistent pipeline. Volumes were resampled to 1 mm isotropic voxel spacing using bilinear interpolation and clipped to an intensity range of $[-1000, 2000]$ Hounsfield units to ensure consistent soft-tissue representation. Intensities were subsequently z-score normalized on a per-patient basis. Pancreas segmentation masks were generated automatically using TotalSegmentator^31^. A fixed-size region of interest (ROI) of $256 \times 256 \times 128$ voxels, centered on the pancreas, was extracted for downstream analysis. To improve robustness to acquisition variability and prevent overfitting, data augmentation was applied during training, including random flips, zooming (0.9–1.2), Gaussian noise ($\sigma = 0.01$), intensity scaling ($\pm 0.3$), and random 90$^\circ$ rotations.

### Model architecture

We employed the CT Vision Foundation Model (CT-FM)^28^, pre-trained on 148,000 volumetric CT scans using contrastive self-supervised learning^29^, as the base architecture for all experiments. For the classical machine learning baselines, the CT-FM encoder was used as a frozen feature extractor. Specifically, 512-dimensional feature embeddings were extracted from the SegResNet backbone and used as input to downstream classifiers. The MLP baseline consisted of a two-layer multilayer perceptron (512–256–2) with ReLU activation and optional dropout for classification, while other machine learning models (e.g., Logistic Regression, LDA, Gradient Boosting, and Random Forest) were trained on the same CT-FM embeddings. In contrast, all PDAC-CTFM models were trained using full end-to-end fine-tuning of the CT-FM architecture for the downstream LNM prediction task. In this setting, both the SegResNet backbone and the classification head were jointly optimized, allowing the representations of the foundation model to adapt to the specific imaging characteristics of PDAC.

### FL framework

FL enables collaborative model training while ensuring that patient data remain within institutional firewalls. At each communication round *t*, the global model parameters $\omega ^{(t)}$ were broadcast to all participating institutions (clients). Each client *k* trained the model locally on its dataset $\mathcal {D}_k$ for 1–3 epochs, yielding updated parameters $\omega _k^{(t+1)}$. Only model weights were transmitted back to the central server, where aggregation was performed to produce the updated global model $\omega ^{(t+1)}$. No raw images, labels, or intermediate activations were shared at any point.

### Heterogeneity-aware feddisco (Ours)

In real-world multi-center medical imaging, non-identically distributed (non-IID) data arise due to differences in scanners, acquisition protocols, reconstruction settings, and patient demographics. Under such conditions, standard federated optimization strategies such as FedAvg^15^ often suffer from client drift and unstable convergence.

FedDisco^30^ partially addresses this challenge by weighting client updates based on label-distribution discrepancies. For each client *k*, the Kullback–Leibler (KL) divergence

$$d_k = \textrm{KL}(D_k \parallel T)$$

quantifies the mismatch between the local empirical label distribution $D_k$ and a target distribution *T*, which was chosen to be uniform in this study.

However, label distributions across institutions may be similar even when their imaging characteristics differ substantially. In such cases, representation-level heterogeneity, rather than label imbalance, becomes the dominant source of performance degradation. While approaches like FedBN^23^ attempt to mitigate this through normalization partitioning, and FedALA^24^ through adaptive local aggregation, neither actively leverages feature divergence to optimize the global model convergence.

*Representation-level discrepancy.* To explicitly capture feature-level divergence across clients, we introduce a model-discrepancy term based on the cosine distance between classifier-layer parameters. Let $\theta _k$ denote the parameters of the final classification layer of client *k*, and let $\theta _{\textrm{ref}}$ correspond to those of the current global model. We define

$$\Delta _k = 1 - \frac{ \langle \theta _k, \theta _{\textrm{ref}} \rangle }{ \Vert \theta _k\Vert \,\Vert \theta _{\textrm{ref}}\Vert }.$$

A larger $\Delta _k$ indicates a decision boundary that deviates more strongly from the global trend, reflecting domain-specific representation drift.

*Unified heterogeneity-aware aggregation.* We combine label-distribution and representation-level discrepancies into a single adaptive aggregation rule. Let $n_k$ denote the number of training samples at client *k*. The unnormalized aggregation weight is defined as

$$p_k \propto \textrm{ReLU}\!\left( n_k - a\, d_k - \gamma \, \Delta _k + b \right) ,$$

where *a* controls sensitivity to label imbalance, $\gamma$ controls sensitivity to representation drift, and *b* is a bias term. The ReLU operator ensures non-negative aggregation weights and prevents unstable contributions from highly divergent clients. Final aggregation weights are obtained via normalization:

$$p_k = \frac{ \textrm{ReLU}\!\left( n_k - a d_k - \gamma \Delta _k + b\right) }{ \sum _{m=1}^{K} \textrm{ReLU}\!\left( n_m - a d_m - \gamma \Delta _m + b\right) }.$$

This formulation preserves the strengths of FedDisco while explicitly addressing feature-level heterogeneity. Unlike optimization-based approaches such as FedProx^32^ or SCAFFOLD^33^, parameter-partitioning strategies like FedBN^23^ or FedALA^24^ that require an auxiliary client-side optimization phase, the proposed method operates entirely at the server aggregation level and requires no architectural modifications or auxiliary control variates.

### Baseline methods

We benchmarked our approach against multiple baselines:

- **Individual baseline**: a CT-FM model fine-tuned separately at each institution (PDAC-CTFM_Individual_) to assess single-site generalization.
- **Classical machine learning baselines**: Logistic Regression, Linear Discriminant Analysis, Kernel SVM, Gradient Boosting, and Random Forest, all trained on CT-FM embeddings using the same training splits as the centralized model.
- **Deep learning baselines**: an MLP classifier and the prior PDAC LNM model by Gaviria *et al.*^14^, which employs a Swin UNETR backbone pre-trained on multi-center abdominal CT data and fine-tuned specifically for LNM detection in PDAC.
- **Federated baselines**:
  - PDAC-CTFM_FedAvg_^15^ (standard federated averaging),
  - PDAC-CTFM_FedDisco_^30^ (label-discrepancy-based weighting),
  - PDAC-CTFM_FedBN_^23^ (batch normalization),
  - PDAC-CTFM_FedALA_^24^ (adaptive local aggregation),
  - PDAC-CTFM_Ours_ (proposed method with label and representation discrepancy).
- **Centralized model**: a CT-FM model fine-tuned on pooled multi-center data (PDAC-CTFM_Central_), serving as an upper-bound reference.

### Evaluation metrics

Model performance was evaluated using Balanced Accuracy (BA), Sensitivity, Specificity, Precision, F1-Score, F2-score, Area Under the ROC Curve (AUC), and Area Under the Precision–Recall Curve (AUPRC). BA and AUPRC were selected as primary metrics due to class imbalance. The Diagnostic Odds Ratio (DOR) was reported for clinical interpretability, as it jointly reflects sensitivity and specificity. Given the clinical importance of minimizing missed metastatic disease, sensitivity-weighted metrics were emphasized while explicitly reporting specificity.

### Implementation details

All preprocessing and data loading were implemented using MONAI^34^. Most models were trained for 100 epochs using the AdamW optimizer (learning rate $10^{-3}$, weight decay 0.01) with cosine annealing scheduling, except for FedBN (learning rate $10^{-3}$, weight decay $10^{-5}$) and FedALA (learning rate $10^{-4}$, weight decay $10^{-5}$). The batch size was 4 with gradient accumulation. Federated training employed 1–3 local epochs per communication round across 20–40 rounds to limit client drift under non-IID conditions while maintaining communication efficiency. Hyperparameters were selected from $a \in \{0.0, 0.1, 0.5, 1.0\}$, $b \in \{0.0, 0.1, 0.5, 1.0\}$, with $\gamma \in \{0, 1, 10\}$ tuned via validation. All experiments were conducted using PyTorch and MONAI on NVIDIA A100 GPUs.

## Results

**Table 1:** Performance comparison of different models on the aggregated test set for LNM detection.

**Model**	**BA**	**Sensitivity**	**Specificity**	**Precision**	**F1 Score**	**DOR**	**AUPRC**
MLP	0.4996	0.6757	0.3235	0.6849	0.6803	0.9964	0.6942
LDA	0.4614	0.5405	0.3823	0.6557	0.5926	0.7283	0.7281
LogRes	0.5358	0.6892	0.3824	0.7083	0.6986	1.3727	0.7015
GB	0.5126	0.8811	0.1441	0.6914	0.7748	1.2646	0.6335
RF	0.4978	0.9162	0.0794	0.6841	0.7833	1.0676	0.6769
Gaviria et al.^14^	0.5000	**1.0000**	0.0000	0.6852	**0.8132**	-	**0.8423**
PDAC-CTFM_FedAvg_	0.4607	0.8919	0.0882	0.6667	0.7630	0.2500	0.6968
PDAC-CTFM_FedDisco_	0.4933	0.1622	0.8235	0.6667	0.2609	0.9032	0.7072
PDAC-CTFM_FedBN_	0.4603	0.0676	**0.8529**	0.5000	0.1190	0.4203	0.6514
PDAC-CTFM_FedALA_	0.5012	0.9730	0.0294	0.6857	0.8045	1.0909	0.7134
PDAC-CTFM_Ours_	0.5866	0.7027	0.4706	**0.7429**	0.7222	2.1010	0.6752
PDAC-CTFM_central_	**0.6010**	0.8784	0.3236	0.7386	0.8025	**3.4541**	0.7376

We evaluate our proposed federated deep learning framework on multi-institutional CT datasets for LNM detection in PDAC. The experiments assess model performance, generalization across centers, and the impact of adaptive aggregation under heterogeneous data conditions (2).

**Fig. 2:**
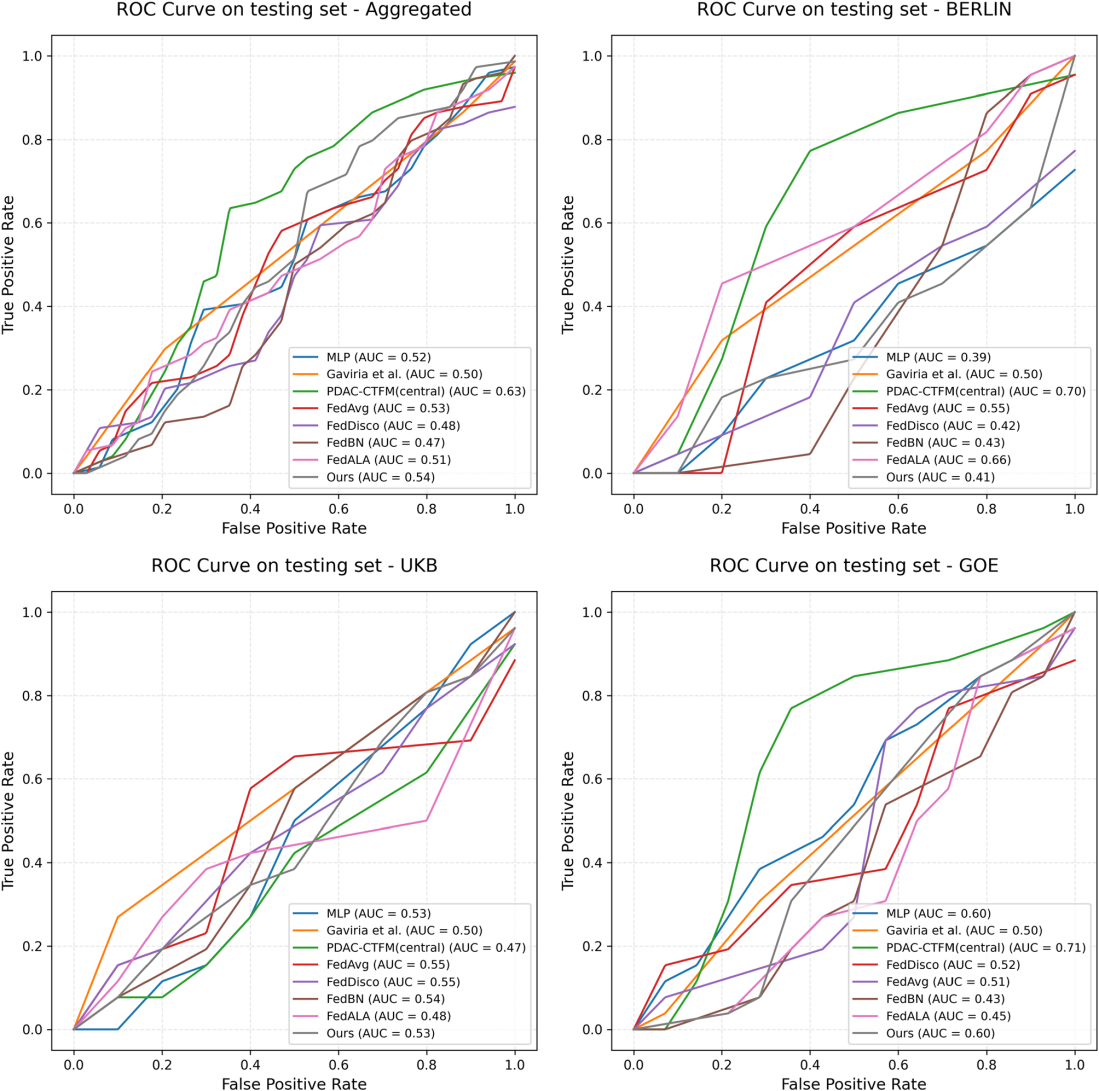
ROC curve comparison of different machine learning models and PDAC-CTFM_central_ and PDAC-CTFM_ours_ across aggregated and site-specific test sets.

### Centralized performance

The centralized fine-tuning of the CT foundation model (PDAC-CTFM_central_) achieved the strongest overall performance across all evaluated models on the aggregated test set of 546 patients (Table 1). With a balanced accuracy (BA) of 0.6010, the model surpassed all classical machine learning baselines, including Logistic Regression (0.5358), Gradient Boosting (0.5126), and Random Forest (0.4978). Compared with our previous PDAC LNM detection model^14^, which achieved a BA of 0.5000, PDAC-CTFM_central_ delivered a substantial absolute improvement of +10.1%.

In addition to balanced accuracy, the centralized model, PDAC-CTFM_central_, demonstrated the highest diagnostic discriminative strength. Its DOR reached 3.4541–the largest among all models–indicating markedly improved ability to distinguish metastatic from non-metastatic cases. This DOR is more than double that of most classical baselines and considerably higher than that of all federated models, reflecting more reliable clinical prediction.

The centralized model also achieved strong class-wise performance: a sensitivity of 0.8784 indicates robust detection of metastatic cases, while its specificity (0.3236), although modest, exceeded that of most classical ML methods and all high-sensitivity baselines. Precision (0.7386) and F1 score (0.8025) were among the highest across all models, demonstrating balanced predictive performance and improved overall reliability. The AUPRC (0.7376) further underscores enhanced separability between metastatic and non-metastatic groups, which is particularly relevant in datasets with moderate class imbalance.

Together, these results show that fine-tuning a large-scale CT foundation model on pooled multi-center data yields superior predictive performance, outperforming both deep learning and classical machine learning methods. This performance sets an upper-bound benchmark for our federated experiments, providing a clear reference for evaluating privacy-preserving alternatives.

### Federated performance

We evaluated several federated variants to assess model performance under decentralized training conditions where patient data remain locally stored at each institution (Table 1). As expected in heterogeneous multi-center datasets, the standard FedAvg approach (PDAC-CTFM_FedAvg_) showed limited robustness, achieving a BA of 0.4607 and a notably low DOR of 0.2500. Although it maintained high sensitivity (0.8919), specificity dropped sharply (0.0882), indicating substantial overfitting to majority class patterns and inability to generalize across centers–consistent with known limitations of FedAvg in non-IID clinical environments.

The original FedDisco variant (PDAC-CTFM_FedDisco_), which adjusts aggregation weights based on label-distribution imbalance, improved specificity substantially (0.8235). However, this came at the cost of severely reduced sensitivity (0.1622), resulting in unstable overall performance (BA = 0.4933). These results suggest that label-discrepancy alone is insufficient to address the deeper representation-level heterogeneity present across sites.

FedBN (PDAC-CTFM_FedBN_) suffered from a trivial prediction collapse under our experimental setting. It classified nearly all instances as the *negative* class, yielding a sensitivity of only 0.0676 and a specificity of 0.8529, with a BA of 0.4603 and a DOR of 0.4203 (Table 1). This negative-prediction bias shows that, without global normalization signals, the local models failed to learn discriminative features for the positive class. This severe instability highlights that merely partitioning architecture parameters is insufficient and can lead to catastrophic failure without active feature-level regularization.

FedALA (PDAC-CTFM_FedALA_)^24^ exhibited the opposite trivial prediction pattern. It classified nearly all instances as the *positive* class, achieving a sensitivity of 0.9730 but a specificity of only 0.0294 (BA = 0.5012, DOR = 1.0909). Although its F1 score appears competitive (0.8045), this is an artifact of the dataset’s moderate class imbalance combined with majority-class prediction. The failure of FedALA in our cross-silo setting is consistent with its original design for large-scale cross-device scenarios with many clients ($\ge$50), where the adaptive aggregation benefits from a richer diversity of local updates rather than in our three-client configuration.

Our proposed heterogeneity-aware federated strategy (PDAC-CTFM_Ours_) achieved the overall best performance among all federated models. It reached a BA of 0.5866, outperforming FedAvg and FedBN substantially by +12.6%, the original FedDisco by +9.3%, and FedALA by +8.5%. PDAC-CTFM_Ours_ also achieved a markedly higher DOR (2.1010), reflecting improved discriminatory ability, and delivered balanced precision (0.7429), sensitivity (0.7027), and specificity (0.4706). These results highlight the importance of incorporating both label-distribution and representation-level discrepancy into the aggregation process.

While the centralized model (BA = 0.6010) still represents the upper-bound scenario, our federated method recovers most of its predictive performance while fully preserving data privacy. The improvement over other baselines demonstrates that explicitly modeling representation heterogeneity provides complementary benefits beyond batch-normalization alignment alone. This outcome emphasizes the feasibility of deploying privacy-preserving, multi-center deep learning systems for PDAC staging in real-world clinical environments.

### Ablation and sensitivity analysis

**Fig. 3:**
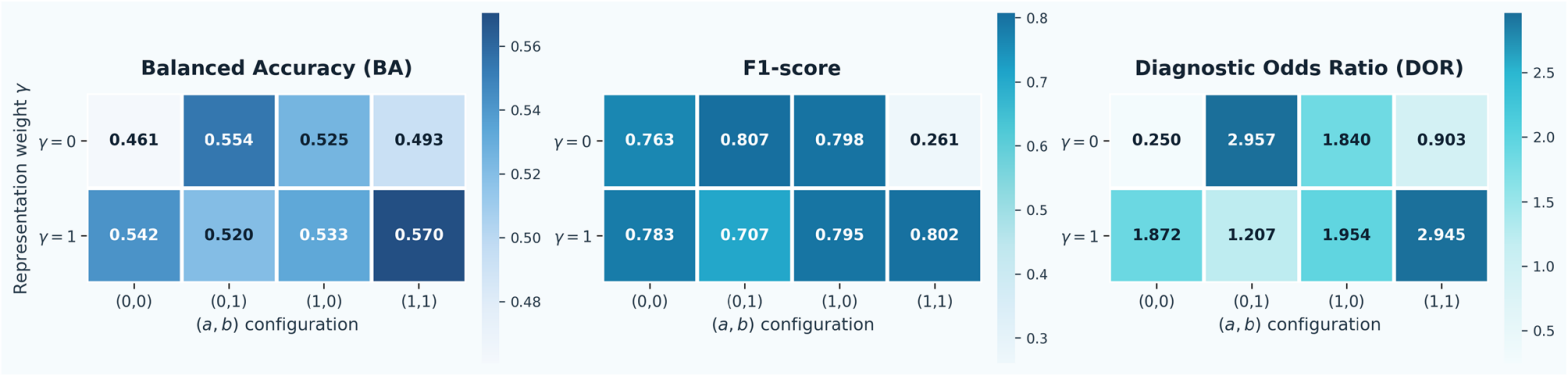
Ablation and sensitivity analysis of the proposed heterogeneity-aware aggregation strategy. Columns correspond to the four (*a*, *b*) configurations and rows to $\gamma \in \{0,1\}$. Each heatmap reports Balanced Accuracy (BA), F1-score, and Diagnostic Odds Ratio (DOR). The results show that incorporating representation-level discrepancy ($\gamma$) improves federated model robustness across heterogeneous centers, with the best overall performance observed for $(a,b,\gamma )=(1,1,1)$.

To analyze the contribution of the proposed heterogeneity-aware aggregation components, we performed an ablation study over the aggregation parameters *a*, *b*, and $\gamma$, which control the influence of label-distribution discrepancy, bias regularization, and representation-level discrepancy, respectively. We evaluated four configurations of $(a,b) \in \{(0,0),(0,1),(1,0),(1,1)\}$ and two values of $\gamma \in \{0,1\}$ to isolate the effect of each component. Figure 3 summarizes the results using heatmaps for Balanced Accuracy (BA), F1-score, and Diagnostic Odds Ratio (DOR). When $\gamma =0$, corresponding to the absence of representation-level discrepancy, performance varies substantially across (*a*, *b*) configurations. In particular, incorporating label-distribution discrepancy (0, 1, 0) improves BA compared with the baseline configuration (0, 0, 0), indicating that accounting for label imbalance partially mitigates cross-client heterogeneity. Introducing representation-level discrepancy ($\gamma =1$) generally improves model robustness. Configurations incorporating both discrepancy terms achieve higher balanced accuracy and diagnostic discriminability, suggesting that combining label-distribution and representation-level alignment leads to more stable aggregation across heterogeneous clinical sites. To determine the final hyperparameter configuration, we additionally conducted a sensitivity analysis on the validation set by exploring smaller discrepancy weights and larger representation penalties. Based on validation performance, we selected $(a,b,\gamma )=(0.1,0.1,10)$ for the final model configuration used in all reported experiments. This setting provided the most balanced trade-off between sensitivity and specificity while maintaining stable performance across centers. The results, therefore, indicate that moderate weighting of both discrepancy terms together with stronger representation-level regularization leads to the most robust federated aggregation.

### Site-wise generalization across clinical centers

The site-specific results (Tables 2, 3,4) reveal substantial variability in model performance across institutions, underscoring the challenges posed by real-world domain heterogeneity in multi-center PDAC imaging. Models trained exclusively on single-site data (PDAC-CTFM_individual_) consistently underperformed across all centers despite leveraging a large-scale CT foundation model. For Berlin and UKB, individual models yielded balanced accuracies of 0.3863 and 0.5000, respectively, with specificity falling to 0.0 in both cases. This behavior indicates severe overfitting to local characteristics and an inability to generalize beyond the institutional domain, thereby reinforcing the necessity of multi-site training for robust LNM prediction.

Classical machine learning baselines exhibited similar limitations, with inconsistent performance across centers. In Berlin, logistic regression achieved the highest BA among classical methods (0.5591), whereas gradient boosting was strongest in UKB (0.5869), and MLP performed best in Göttingen (0.5604). However, none of these approaches exhibited stable performance across datasets, and DOR values generally remained low, reflecting limited clinical discriminability. This inconsistency highlights the inadequacy of traditional models under heterogeneous imaging distributions.

By contrast, the centralized PDAC-CTFM_central_ model demonstrated strong cross-site generalization. It achieved BA values of 0.5540 (Berlin), 0.6923 (Göttingen), and 0.5230 (UKB), consistently outperforming both individual models and prior work^14^. The Göttingen cohort showed particularly notable gains, with a DOR of 7.6660–the highest observed across all experiments–indicating excellent diagnostic separability in a previously unseen domain. This improvement illustrates the value of large-scale pre-training combined with pooled multi-center fine-tuning.

The FL experiments further reveal distinct patterns across sites. FedAvg produced unstable behavior, frequently collapsing specificity to 0.0 (Berlin and UKB) despite high sensitivity. This imbalance resulted in poor BA values (0.4773, 0.4423) and DOR values of 0, demonstrating FedAvg’s vulnerability to non-IID distributions. The original FedDisco variant improved specificity in Berlin (0.7000) and Göttingen (0.7857), but at the cost of severely reduced sensitivity (0.1364 and 0.3077, respectively), yielding only marginal gains in overall performance.

FedBN exhibited consistent negative-prediction bias across all three centers, with sensitivity values of 0.0455 (Berlin), 0.0769 (Göttingen), and 0.0769 (UKB), while maintaining high specificity (0.9000, 0.7857, and 0.9000, respectively). The resulting BA values (0.4727, 0.4313, 0.4885) and uniformly low DOR values confirm that the trivial prediction collapse observed on the aggregated test set was not site-specific but rather a systematic failure of the normalization-partitioning strategy under the heterogeneity conditions of these cohorts.

Conversely, FedALA collapsed to near-universal positive prediction across centers, with sensitivity values of 1.0000 (Berlin), 0.9615 (Göttingen), and 0.9615 (UKB), while specificity dropped to at most 0.1000, respectively. This pattern mirrors the FedAvg failure mode and indicates that the client-side phase to learn local aggregation weights alone is insufficient when the number of participating clients is small and their data distributions differ substantially.

**Fig. 4:**
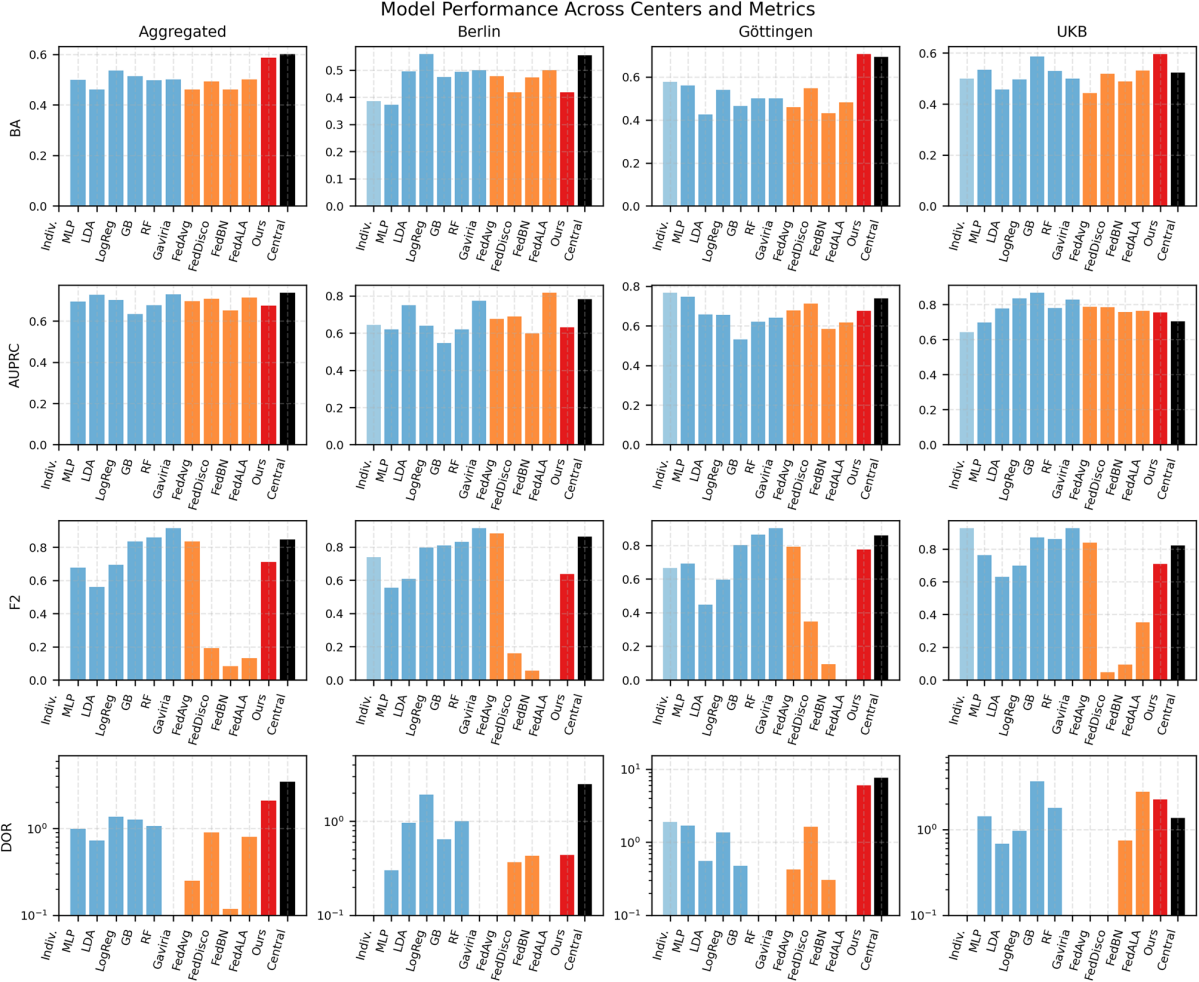
Comparison of model performance across four evaluation metrics (Balanced Accuracy, AUPRC, F2-score, and Diagnostic Odds Ratio) for aggregated and site-specific test sets (Berlin, Göttingen, UKB). Rows correspond to evaluation metrics, and columns correspond to clinical centers. Each subplot reports performance for classical machine learning models, federated baselines, the individual and centralized CT-FM models, and the proposed heterogeneity-aware federated method. The figure summarizes inter-model variability, highlights site-specific domain effects, and illustrates the performance gains achieved by the proposed federated strategy.

Our proposed PDAC-CTFM_Ours_ method provided the most balanced and robust federated performance across centers. It produced BA values of 0.4182 (Berlin), 0.7060 (Göttingen), and 0.5962 (UKB), outperforming all federated methods in Göttingen and UKB. Importantly, it maintained well-balanced sensitivity and specificity profiles, leading to substantially higher DOR values–particularly in Göttingen (6.0000) and UKB (2.2500). These results demonstrate the advantage of incorporating both label-distribution discrepancy and representation-level cosine distance into the aggregation process. This dual-discrepancy strategy enables improved alignment of decision boundaries across centers, enhancing the global model’s ability to generalize to diverse institutional domains (Fig. 4).

Together, these findings highlight three key insights: (1) Single-site models fail to generalize even when built on strong foundation model encoders. (2) Pooled multi-center training remains the strongest paradigm but is often impractical due to privacy constraints. (3) Our federated strategy recovers much of the centralized model’s performance while preserving data locality, offering a viable path toward equitable and scalable deployment of LNM detection tools in real-world clinical networks (3).

**Table 2:** Performance of different models on the Berlin test set.

**Model**	**BA**	**Sensitivity**	**Specificity**	**Precision**	**F1 Score**	**DOR**	**AUPRC**
PDAC-CTFM_individual_	0.3863	0.7727	0.0000	0.6296	0.6938	0.0000	0.6442
MLP	0.3727	0.5455	0.1999	0.6000	0.5714	0.3000	0.6192
LDA	0.4955	0.5909	0.4001	0.6842	0.6341	0.9630	0.7502
LogRes	**0.5591**	0.8182	0.3000	**0.7200**	0.7660	1.9286	0.6396
GB	0.4750	0.8500	0.1000	0.6750	0.7524	0.6426	0.5472
RF	0.4936	0.8773	0.1099	0.6842	0.7687	1.0056	0.6202
Gaviria et al.^14^	0.5000	**1.0000**	0.0000	0.6875	**0.8148**	-	**0.8437**
PDAC-CTFM_FedAvg_	0.4773	0.9545	0.0000	0.6774	0.7925	0.0000	0.6767
PDAC-CTFM_FedDisco_	0.4182	0.1364	0.7000	0.5000	0.2143	0.3684	0.6893
PDAC-CTFM_FedBN_	0.4727	0.0455	**0.9000**	0.5000	0.0833	0.4286	0.5987
PDAC-CTFM_FedALA_	0.5000	**1.0000**	0.0000	0.6875	**0.8148**	0.0000	0.8172
PDAC-CTFM_Ours_	0.4182	0.6364	0.2000	0.6364	0.6364	0.4375	0.6314
PDAC-CTFM_central_	0.5540	0.9091	0.1989	0.7143	0.8000	**2.5000**	0.7823

**Table 3:** Performance of different models on the Göttingen test set.

**Model**	**BA**	**Sensitivity**	**Specificity**	**Precision**	**F1 Score**	**DOR**	**AUPRC**
PDAC-CTFM_individual_	0.5769	0.6538	0.5000	0.7083	0.6800	1.8888	0.7672
MLP	0.5604	0.6923	0.4285	0.6923	0.6923	1.6875	0.7466
LDA	0.4258	0.4231	0.4285	0.5789	0.4889	0.5500	0.6570
LogRes	0.5385	0.5769	0.5001	0.6818	0.6250	1.3630	0.6552
GB	0.4646	0.8577	0.0715	0.6317	0.7275	0.4731	0.5310
RF	0.4997	0.9423	0.0571	0.6496	0.7690	$\infty$	0.6199
Gaviria et al.^14^	0.5000	**1.0000**	0.0000	0.6500	0.7878	-	**0.8250**
PDAC-CTFM_FedAvg_	0.4588	0.8462	0.0714	0.6286	0.7213	0.4231	0.6768
PDAC-CTFM_FedDisco_	0.5467	0.3077	**0.7857**	0.7273	0.4324	1.6296	0.7121
PDAC-CTFM_FedBN_	0.4313	0.0769	**0.7857**	0.4000	0.1290	0.3056	0.5840
PDAC-CTFM_FedALA_	0.4808	0.9615	0.0000	0.6410	0.7692	0.0000	0.6160
PDAC-CTFM_Ours_	**0.7060**	0.7692	0.6429	**0.8000**	0.7843	6.0000	0.6759
PDAC-CTFM_central_	0.6923	0.8846	0.5000	0.7666	**0.8214**	**7.6660**	0.7385

**Table 4:** Performance of different models on the UKB test set.

**Model**	**BA**	**Sensitivity**	**Specificity**	**Precision**	**F1 Score**	**DOR**	**AUPRC**
PDAC-CTFM_individual_	0.5000	**1.0000**	0.0000	0.7222	**0.8387**	0.0000	0.6411
MLP	0.5346	0.7692	0.3000	0.7407	0.7547	1.4286	0.6969
LDA	0.4577	0.6154	0.3000	0.6957	0.6531	0.6857	0.7774
LogRes	0.4962	0.6923	0.3001	0.7200	0.7059	0.9643	0.8354
GB	0.5869	0.9038	0.2700	0.7631	0.8274	**3.6488**	**0.8677**
RF	0.5300	0.9000	0.1600	0.7360	0.8096	1.7977	0.7798
Gaviria et al.^14^	0.5000	**1.0000**	0.0000	0.7222	**0.8387**	-	0.8611
PDAC-CTFM_FedAvg_	0.4423	0.8846	0.0000	0.6970	0.7797	0.0000	0.7863
PDAC-CTFM_FedDisco_	0.5192	0.0385	**1.0000**	**1.0000**	0.0741	0.0000	0.7853
PDAC-CTFM_FedBN_	0.4885	0.0769	0.9000	0.6667	0.1379	0.7500	0.7578
PDAC-CTFM_FedALA_	0.5308	0.9615	0.1000	0.7353	0.8333	2.7778	0.7646
PDAC-CTFM_Ours_	**0.5962**	0.6923	0.5000	0.7826	0.7347	2.2500	0.7552
PDAC-CTFM_central_	0.5230	0.8462	0.1998	0.7333	0.7857	1.3750	0.7039

**Fig. 5:**
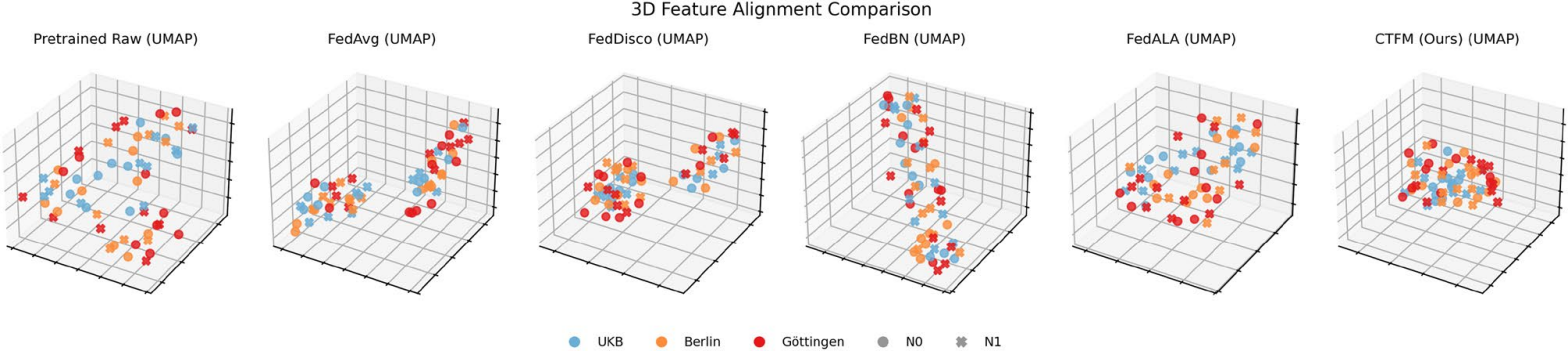
UMAP visualizations of feature representations from the three clinical centers (Berlin, Göttingen, and UKB). (a) The raw features from the frozen CT-FM backbone exhibit distinct site-specific clustering, indicating a dominant domain shift. (b) In contrast, our proposed heterogeneity-aware aggregation effectively ’blends’ these distributions, fostering a more integrated and domain-invariant representation space. This visual alignment substantiates our method’s ability to filter out acquisition-related noise and focus on pathological signals relevant to LNM detection.

**Fig. 6:**
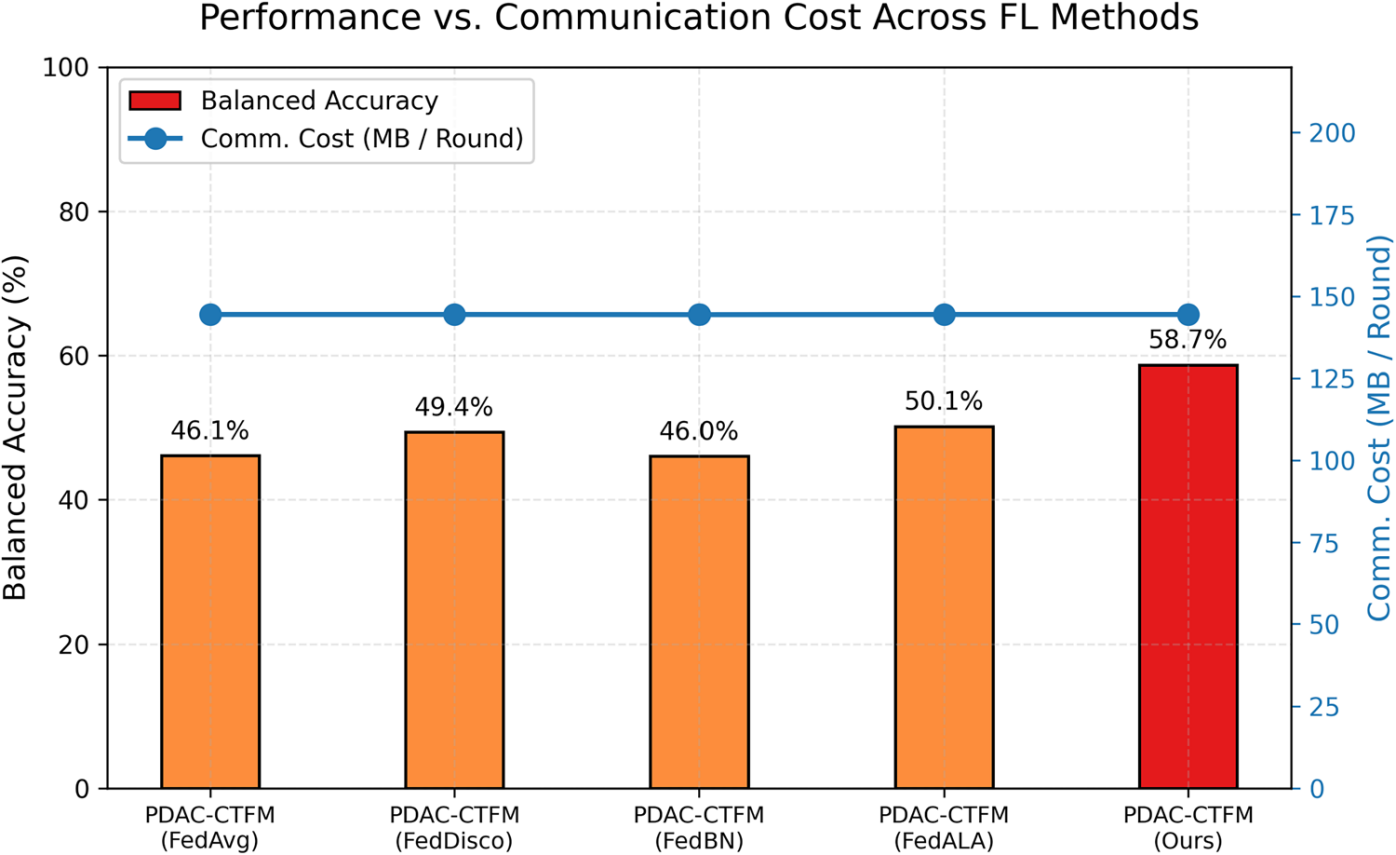
Performance vs. Communication Cost across evaluated federated learning methods. All methods utilize the same CT-FM backbone ($\approx 18.9$M parameters), resulting in an identical communication payload of 144.44 MB per round (144.39 MB for FedBN). The secondary y-axis (red) illustrates the communication cost, while the primary y-axis (bars) reports the achieved Balanced Accuracy. Our proposed method (PDAC-CTFM) introduces negligible server-side computational overhead ($<0.01$s) while recovering substantially higher diagnostic performance.

## Discussion

### Central findings and clinical implications

This study demonstrates that integrating a large-scale CT foundation model pre-training with heterogeneity-aware federated optimization substantially improves LNM detection in PDAC. Across all experimental settings, the centralized PDAC-CTFM model consistently outperformed classical machine learning approaches, previously published PDAC LNM predictors, and all federated variants. These results highlight the strong capacity of foundation models, pre-trained on large and diverse CT corpora, to extract domain-robust representations that capture subtle imaging patterns associated with metastatic spread.

From a clinical perspective, the observed improvements in balanced accuracy and diagnostic odds ratio (DOR) are particularly relevant. DOR jointly reflects sensitivity and specificity and is commonly interpreted as a global indicator of diagnostic usefulness. The high DOR values achieved by the centralized model, especially in the Göttingen cohort, suggest improved discriminative power that could meaningfully support preoperative staging and treatment planning. Importantly, these gains were achieved without reliance on handcrafted radiomic features or explicit lymph node annotations, underscoring the scalability and practicality of the proposed approach.

### Generalization and cross-center heterogeneity

A key observation in this study is the limited generalization of models trained on single-center data. Even when initialized with a strong foundation model encoder, site-specific models exhibited marked performance degradation when evaluated on external cohorts, often manifesting as sharp declines in balanced accuracy and near-zero specificity. This behavior reflects severe overfitting to institution-specific imaging characteristics. It reinforces a long-standing challenge in medical AI: models trained in isolation rarely transfer reliably across centers with differing scanners, acquisition protocols, and patient populations.

The strong performance of the centralized PDAC-CTFM model, trained on pooled multi-center data, highlights the value of exposure to heterogeneous imaging distributions during training. However, such centralized aggregation is rarely feasible in clinical practice due to regulatory, ethical, and logistical constraints. The pronounced performance differences observed across Berlin, Göttingen, and UKB further emphasize that cohort-specific factors, such as contrast timing, reconstruction kernels, and referral patterns, can substantially influence model behavior.

These observations align with recent findings reported by Zheng et al.^13^, who highlighted that performance gains are often strongly influenced by dataset-specific characteristics and can degrade substantially under cross-cohort distribution shifts. Their analysis emphasizes that high performance on internal test sets does not necessarily translate to robust generalization across institutions, even for models trained on large datasets. Our results provide complementary empirical evidence of this challenge in the context of multi-center PDAC CT imaging and further motivate the need for training strategies that explicitly address cross-domain heterogeneity.

### Clinical impact of moderate specificity

Across all models, including the best-performing centralized and federated variants, specificity remained moderate compared to sensitivity. Specifically, the centralized model achieved a specificity of 0.3236, while our federated approach improved this to 0.4706 on the aggregated test set. In clinical practice, maintaining a sensitivity of 0.7027 while improving specificity results in a non-negligible false-positive rate, which, if used in isolation, could lead to over-staging and potentially unnecessary neoadjuvant therapy or extended surgical explorations.

However, in PDAC staging, sensitivity is systematically prioritized over specificity: missing a metastatic lymph node carries severe prognostic consequences, including inadequate surgical planning and suboptimal therapy selection^3^. The clinical literature reports that CT-based assessment of LNM exhibits poor sensitivity (14–44%) under standard radiological criteria^4,5^. Although the moderate specificity observed in our models improves by approximately 3%, it also highlights the inherent challenge of PDAC-LNM.

Additionally, the proposed model is designed as a *decision-support tool* to complement, rather than replace, radiological assessment and multidisciplinary tumor board consensus. In clinical workflows, an over-prediction of LNM would prompt secondary diagnostic workups or closer intraoperative staging, safely bounding the risk of over-treatment while maximizing the detection of LNM.

### FL under real-world constraints

FL offers a principled mechanism to enable multi-center collaboration without centralizing sensitive patient data. However, our results confirm that naïve FL strategies struggle under realistic clinical heterogeneity. Standard FedAvg exhibited unstable behavior, frequently collapsing specificity while maintaining high sensitivity, leading to poor balanced accuracy and limited clinical utility. This outcome aligns with prior reports describing FedAvg’s vulnerability to non-IID data distributions in medical imaging.

The original FedDisco algorithm, which reweights clients based on label-distribution discrepancy, provided partial improvements but remained insufficient in this setting. In several cohorts, FedDisco produced extreme trade-offs between sensitivity and specificity, suggesting that label imbalance alone does not adequately capture the dominant sources of heterogeneity in multi-center CT data. Feature-level and representational differences appear to play a more critical role.

Notably, FedBN^23^ suffered from model collapse in our setting, predicting all test cases as LNM-negative. Conversely, FedALA^24^ exhibited the opposite collapse pattern, predicting nearly all cases as LNM-positive. These complementary failure modes underscore that purely architectural parameter-partitioning strategies and client-side optimization are insufficient under the severe label and feature heterogeneity present in our three-center PDAC cohort. While these approaches offer complementary perspectives, our results demonstrate that explicitly modeling representation-level discrepancy at the aggregation level provides a direct and effective solution for the domain-shift challenge in multi-center medical imaging.

Our proposed extension of FedDisco addresses this limitation by incorporating a representation-level discrepancy term based on the cosine distance between client-specific and global decision boundaries. By explicitly down-weighting clients whose learned representations diverge substantially from the global trend, the aggregation process becomes more stable and better aligned across centers. This strategy yielded the strongest performance among all federated approaches and recovered a substantial fraction of the centralized model’s accuracy while preserving strict data privacy. The associated gains in DOR further indicate improved diagnostic reliability, strengthening the clinical relevance of representation-aware aggregation in federated medical imaging. This alignment is visually substantiated by the UMAP embeddings (Fig. 5). In the challenging task of LNM detection in CT scans, site-specific acquisition parameters often introduce a domain shift that is more pronounced than the subtle pathological signal itself. While most baseline methods exhibit distinct ’domain islands’–where samples group primarily by two to three clusters—our proposed PDAC-CTFM yields a more integrated and homogeneous representation across Berlin, Göttingen, and UKB. By effectively ’blending’ these distributions, our method filters out site-specific acquisition noise, allowing the model to focus on the underlying features relevant to LNM detection. These qualitative results demonstrate a reduction in representation-level domain shift, successfully bridging the gap between isolated clinical silos.

### Computational and communication efficiency

A critical consideration for deploying FL in real-world clinical networks is the associated computational and communication overhead. Because all evaluated federated methods (FedAvg, FedDisco, FedBN, FedALA, and Ours) share the same foundation model backbone (CT-FM SegResNet) and classification head, the communication payload per round is effectively identical across all implementations. Specifically, the model comprises approximately 18.9 million parameters and requires an uplink and downlink payload of exactly 144.44 MB per client per round (using 32-bit floats).

Crucially, our proposed representation-level discrepancy aggregation adds negligible computational overhead. While the local training complexity per client is structurally matched across standard FL baselines (requiring identical forward and backward passes), our server-side aggregation operates only on the final fully connected classifier head (514 parameters) and on local label distribution statistics. Computing the cosine distance and KL divergence on this lightweight metadata incurs less than 2 KB of communication per round (0.001% overhead). It requires negligible additional computational latency at the central server (<0.01 seconds). This demonstrates that the substantial performance gains achieved by explicitly modeling representational drift do not compromise the scalability or bandwidth efficiency required for distributed healthcare systems, as summarized in Fig. 6.

### Limitations and future directions

Despite these advances, several limitations warrant discussion. First, in all models, including the best-performing centralized and federated variants, specificity remained relatively low compared to sensitivity. Clinically, this reflects a tendency to over-predict LNM, which could lead to overtreatment if used in isolation. However, in the staging of PDAC, sensitivity is often prioritized to avoid missing metastatic disease, which has major prognostic implications. Improving specificity will likely require complementary strategies, such as multi-instance modeling of individual lymph nodes, explicit calibration objectives, or integration of clinical biomarkers (e.g., CA19-9 levels).

Second, while foundation model initialization significantly improves sample efficiency and robustness, federated training introduces non-trivial computational overhead. Communication costs and repeated local optimization may limit accessibility for institutions with constrained computational resources. More efficient parameter adaptation strategies, such as low-rank adaptation (LoRA) or other lightweight adapter modules, represent a promising direction for future work. Such approaches could reduce overfitting, lower communication costs, and enable more efficient personalization without modifying the entire backbone network.

Finally, this study remains retrospective and imaging-only. Prospective validation and integration of multimodal clinical data^35^ will be essential steps toward real-world deployment. Additionally, harmonization techniques and domain-adaptive calibration strategies may further reduce residual inter-center variability and improve reliability across diverse clinical environments.

In summary, this work demonstrates that foundation model transfer learning and heterogeneity-aware federated optimization act synergistically to enable robust, generalizable, and privacy-preserving LNM detection in PDAC. By explicitly addressing both statistical and representation-level heterogeneity, the proposed framework bridges the gap between centralized performance and decentralized feasibility. These findings provide a scalable pathway toward deploying clinically meaningful deep learning systems across distributed healthcare networks and contribute to the broader goal of reliable multi-center medical AI.

## Conclusion

This study presents a multicenter, privacy-preserving framework for LNM detection in PDAC that integrates large-scale CT foundation-model pretraining with heterogeneity-aware federated optimization. The centralized PDAC-CTFM model achieved strong and consistent performance across three independent clinical cohorts, demonstrating the value of foundation models for learning transferable radiological representations from heterogeneous CT data.

Under data-sharing constraints, the proposed FL strategy recovered a substantial proportion of the centralized model’s performance while ensuring that all patient data remained local to each institution. By jointly accounting for label-distribution imbalance and representation-level divergence during aggregation, the framework exhibited improved robustness and diagnostic discriminability compared with standard federated baselines such as FedAvg, FedDisco, FedBN, and FedALA. These findings emphasize that addressing both statistical and feature-level heterogeneity is critical for effective FL in real-world medical imaging applications.

Overall, this work demonstrates that combining foundation-model pretraining with discrepancy-aware federated optimization provides a scalable, clinically relevant pathway for multicenter PDAC staging without compromising data privacy. Future work will focus on integrating complementary clinical variables, developing advanced harmonization and calibration strategies to improve specificity further, and conducting prospective evaluations to assess clinical utility in routine workflows.

## Data Availability

The clinical CT datasets used in this study were obtained from three participating institutions (University Hospital Bonn, Charité-Universitätsmedizin Berlin, and University Medicine Göttingen). Due to institutional data protection policies, ethical restrictions, and GDPR governing patient privacy, the raw imaging data cannot be publicly shared. De-identified derived data supporting the findings of this study, including aggregated model outputs, evaluation metrics, and trained model weights for the centralized and federated configurations, are available from the corresponding author upon reasonable request and subject to institutional approval and data-sharing agreements. The code is publicly available at: <span fontcategory=“NonProportional” name=“Emphasis” class=“NonProportional”>https://github.com/albarqounilab/PDAC-CTFM.
